# Bowel volvulus in the chest after an esophagectomy: an uncommon type IV hiatal hernia

**DOI:** 10.1093/jscr/rjad167

**Published:** 2023-04-12

**Authors:** Danny Ludena, Rodolfo Camillo, Mayara Machry, Paola Solis-Pazmino

**Affiliations:** Surgery Department, Santa Casa de Misericórdia in Porto Alegre (SCMPA), Porto Alegre, RS, Brazil; Surgery Department, Santa Casa de Misericórdia in Porto Alegre (SCMPA), Porto Alegre, RS, Brazil; Surgery Department, Santa Casa de Misericórdia in Porto Alegre (SCMPA), Porto Alegre, RS, Brazil; Surgery Department, Santa Casa de Misericórdia in Porto Alegre (SCMPA), Porto Alegre, RS, Brazil

## Abstract

Transverse colon volvulus is an uncommon cause of bowel obstruction. Moreover, a thoracic herniation into the thorax is still rare. An early diagnosis and treatment are critical to the patient since they can lead to bowel infarction, peritonitis and death. We reported a 55-year-old woman admitted to the emergency department at a hospital. She presented with severe abdominal pain, mainly in the epigastrium, associated with dyspnea, nausea and vomiting. An abdominal CT scan showed a large hiatal hernia in the thorax with signs of volvulus in the involved segment.

## INTRODUCTION

Type IV hiatal hernia is associated with a large defect that can allow other organs, such as the colon, spleen and pancreas, to herniate. It is an uncommon disease with an incidence of 5–15% of all hiatal hernias [1]. The etiology is still unclear, and the spectrum of symptoms, ranging from none to an acute incarcerated hernia, makes a challenging diagnosis [2].

Type IV hernia can be associated with severe complications such as intrathoracic incarceration of the stomach, bleeding, perforation and intestinal volvulus. The volvulus can commonly appear in the sigmoid (60–75%) and the cecum (25–40%), being unusual at the level of the transverse colon [3].

The optimal surgical approach is controversial between open and laparoscopic surgery. Some studies support that the laparoscopic technique is a safe and feasible technique. Other authors prefer open techniques based on clinical experience [4].

We reported a 55-year-old woman admitted to the emergency department who presented with a type IV hiatal hernia into the thorax with a successful treatment after an emergency operation.

## CASE REPORT

A 55-year-old woman with a medical history of arterial hypertension, anterior communicating artery aneurysm with clipping and an esophageal adenocarcinoma underwent ChemoRadiotherapy for Oesophageal cancer followed by Surgery Study. At admission, the patient had stable and normal vital signs, with acute dyspnea, nausea and vomits. She referred to chronic abdominal pain (15 days) with exacerbation in the last hours. On physical exam, she reported diffuse pain on palpation focused on the epigastrium, without peritonism and a negative Murphy’s sign. Cardiorespiratory auscultation was normal.

**Figure 1:**
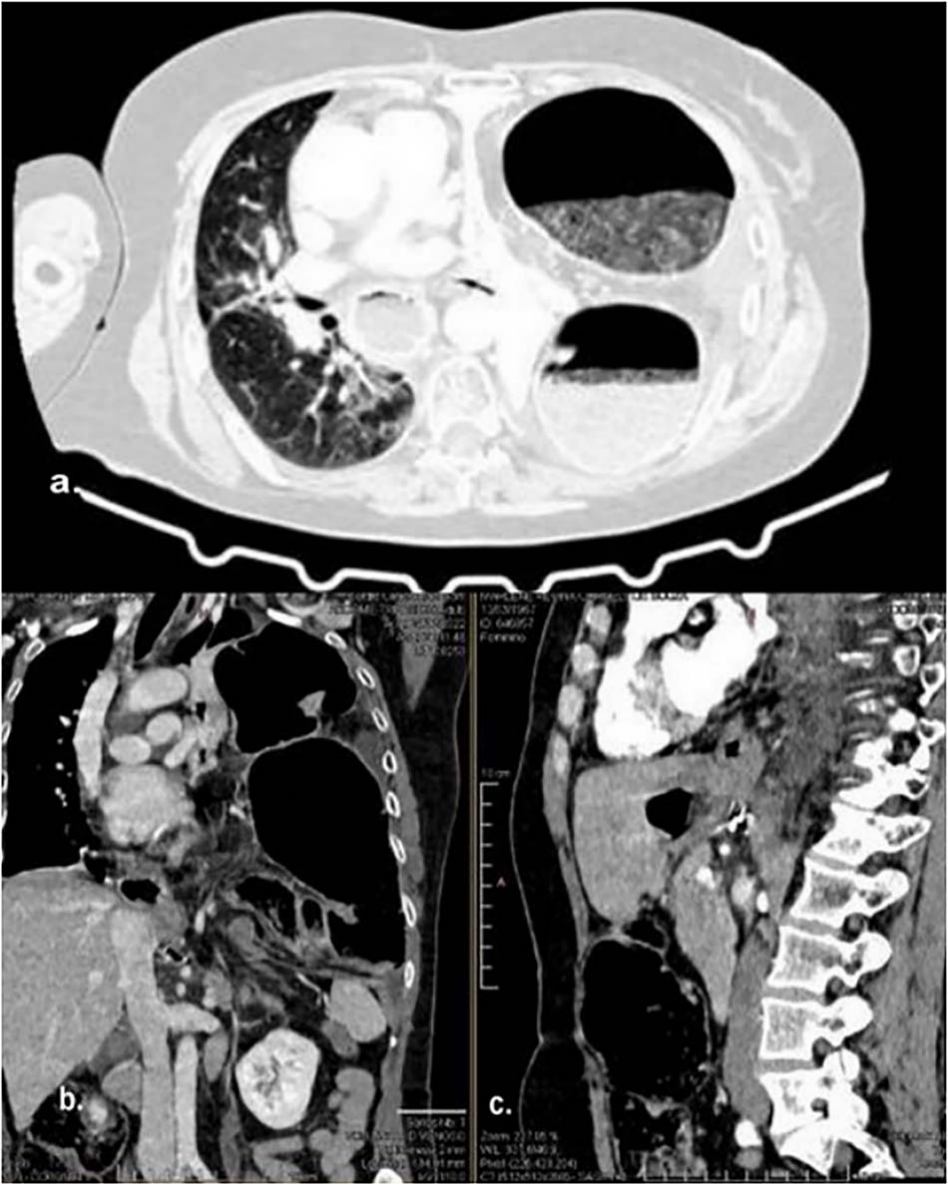
Different sections of the CT study demonstrate the mediastinal position of the transversal volvulus. (**a)** Axial CT section of the abdomen showing signs of esophagectomy with gastric lifting, a type IV hiatal hernia and colon herniation into the thorax. (**b)** The coronal CT section of colon herniation into the thorax. (**c)** The sagittal CT section of the thorax and the abdomen shows the posterior mediastinal position of the colon herniation into the thorax.

The abdominal computed tomography (CT) scan exhibited signs of esophagectomy with gastric lifting, a type IV hiatal hernia and colon herniation into the thorax (Fig. 1a and b). This was full of feces and symptoms of volvulus in the involved segment, with no free liquid in the cavity. Laboratory testing revealed an elevated protein C-reactive of 77, with normal white blood cells. Renal function and electrolytes were within normal limits.

### Treatment

**Figure 2:**
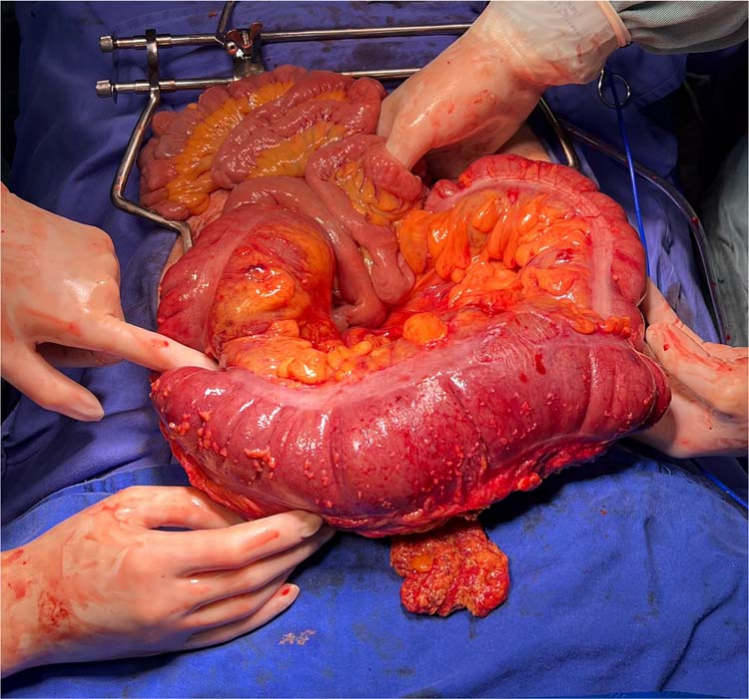
The intraoperative revealed colon herniation through the diaphragmatic hiatus, and the transverse colon appeared distended with erosions in the serosa.

Initial treatment included volume replacement of crystalloids (Ringer lactate), correction of electrolyte disturbances, analgesia and antibiotic therapy. Then we performed an urgent exploratory laparotomy. The intraoperative revealed multiple adhesions of the gastric tube with the left hepatic segment, greater omentum and colon herniation through the diaphragmatic hiatus through a 30 mm hole. From the hiatus, it was possible to reduce the intestinal segment (transverse colon), which appeared distended and with erosions in the serosa (Fig. 2), but without perforation or signs of ischemia. Finally, it was necessary to perform a hiatoplasty to reconstruct the local anatomy.

### Follow-up

**Figure 3:**
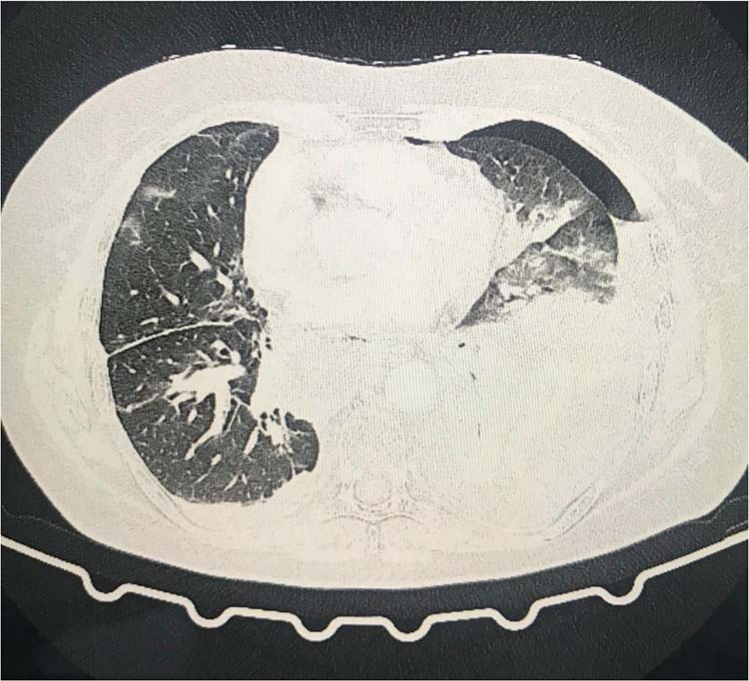
Second day follow-up showed a hydropneumothorax on the left chest CT scan.

**Figure 4:**
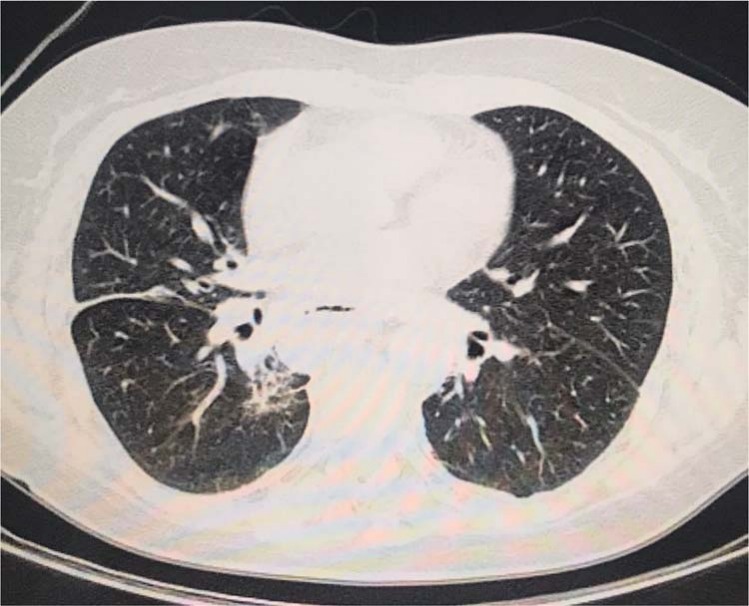
Normal chest CT scan.

On the second day postoperatively, the patient presented with dyspnea and fatigue; a new chest CT evidenced a hydropneumothorax on the left (Fig. 3). Water-seal chest drainage was performed, with an adequate re-expansion despite the chronicity of the hernia and improvement in ventilatory parameters and symptoms (Fig. 4). She was discharged after 9 days of hospitalization. Currently, she is doing well with a satisfactory response to treatment.

### Differential diagnosis

The initial suspicion was an acute obstructive abdomen caused as a first option by the hiatal hernia. The patient was already diagnosed with this hernia and was in preparation for elective surgery. We already had this information when we received her in the emergency room.

Another differential diagnosis was an intestinal obstruction by adhesions. We thought it was this differential due to the history of a past surgery (esophagectomy). It may occur as early as a few weeks or as late as several years after surgery without any prominent inciting event.

## DISCUSSION

Transverse volvulus is a rare disease uncommonly considered the initial underlying cause of abdominal pain and can have a missed or delayed diagnosis and treatment. We presented a middle-aged woman with an unexpected hiatal hernia in the thorax with signs of volvulus in the involved segment.

Paraoesophageal hernias are mainly affecting the older adults. The herniation of the stomach characterizes it along with associated viscera through the esophageal hiatus [5]. Although the etiology is unknown, some predisposing factors include congenital, physiological and acquired [6]. The last include distal colonic obstruction, adhesions, inflammatory strictures, carcinoma and malposition of the colon following previous surgery. It can be combined with an enlarged diaphragmatic hiatus [3]. In a retrospective review of 2182 esophagectomy patients, the risk of a hiatal hernia was <1%, with a higher rate after minimally invasive esophagectomy (MIE) compared with an open approach (2.8 vs. 0.8%). Our patient had an MIE due to adenocarcinoma.

Type IV is associated with a large defect in the phrenoesophageal membrane, requiring emergent repair in patients with acute gastric or colonic volvulus. It avoids complications such as bleeding, obstruction, strangulation, perforation or secondary respiratory compromise associated with high mortality rates [7]. The optimal surgical approach is controversial between open and laparoscopic surgery. Some studies support the laparoscopic technique as a safe and feasible technique. Other authors prefer open techniques based on clinical experience [8]. Our patient was treated with an open procedure due to the acute presentation and the complexity of the intestinal obstruction.

## CONCLUSION

Transverse colon volvulus is an uncommon disease. Its early diagnosis is challenging and allows an emergency treatment to guarantee the wellness of the patient and a successful outcome.
